# The importance of greater speed in drug development for advanced malignancies

**DOI:** 10.1002/cam4.1454

**Published:** 2018-03-30

**Authors:** David J. Stewart, Andrew A. Stewart, Paul Wheatley‐Price, Gerald Batist, Hagop M. Kantarjian, Joan Schiller, Mark Clemons, John‐Peter Bradford, Laurel Gillespie, Razelle Kurzrock

**Affiliations:** ^1^ The University of Ottawa Ottawa Ontario Canada; ^2^ The Ottawa Hospital Ottawa Ontario Canada; ^3^ CareMedics Manotick Ontario Canada; ^4^ Segal Cancer Centre Jewish General Hospital McGill University Montreal Quebec Canada; ^5^ University of Texas MD Anderson Cancer Center Houston Texas; ^6^ The Inova Dwight and Martha Schar Cancer Institute Fairfax, Virginia and Lung Cancer Research Foundation New York USA; ^7^ Bradford Bachinski Limited and the Life Saving Therapies Network Ottawa Ontario Canada; ^8^ University of California San Diego Moores Cancer Center San Diego California

**Keywords:** Cancer, drug approval, drug development, life‐years saved

## Abstract

It takes on average 6–12 years to develop new anticancer drugs from discovery to approval. Effective new agents prolong survival. To demonstrate the importance of rapid drug approval, we calculated life‐years potentially saved if selected agents were approved more rapidly. As illustrative examples, we used 27 trials documenting improvements in survival. We multiplied improvement in median survival by numbers of patients dying annually and multiplied this by number of years from drug discovery until approval. For every year by which time to drug approval could have been shortened, there would have been a median number of life‐years potentially saved of 79,920 worldwide per drug. Median number of life‐years lost between time of drug discovery and approval was 1,020,900 per example. If we were able to use available opportunities to decrease the time required to take a drug from discovery to approval to 5 years, the median number of life‐years saved per example would have been 523,890 worldwide. Various publications have identified opportunities to speed drug development without sacrificing patient safety. While many investigational drugs prove to be ineffective, some significantly prolong survival and/or reduce suffering. These illustrative examples suggest that a substantial number of life‐years could potentially be saved by increasing the efficiency of development of new drugs for advanced malignancies.

## Introduction

For new anticancer agents, the average time from drug discovery to marketing approval was 8 years in the 1960s 1, but had increased to 13.9 years from 2000 onwards 2, prior to recently decreasing for some drugs 3. With “breakthrough drug” legislation 4, the US Food and Drug Administration (FDA) recently granted rapid approval for several promising new agents based on high response rates in phase I‐II trials without requiring the time and expense of phase III trials 5, 6, 7. For example, crizotinib and ceritinib gained accelerated approval for use in patients with ALK‐rearranged nonsmall cell lung cancer (NSCLC) in 6.0 and 6.4 years, respectively, from date of patent application 8, 9, 10, 11.

## Potential Gains from Speeding Drug Development Processes

Several factors contribute to long time intervals between drug discovery and approval, and there are opportunities to reduce this time interval without sacrificing patient safety or data integrity 12, 13, 14, 15, 16, 17. In addition, even if an effective new drug is approved for marketing, there can be long delays in some jurisdictions before payers agree to provide the drug to patients 18, 19, 20. Reducing the time required for making new drugs available to relevant patients is of high importance, as it could reduce suffering while translating into a substantial number of life‐years saved. To illustrate this, we took as examples selected new therapies that had undergone phase III clinical trials published between 2001 and 2015 21, 22, 23, 24, 25, 26, 27, 28, 29, 30, 31, 32, 33, 34, 35, 36, 37, 38, 39, 40, 41, 42, 43, 44, 45, 46, 47, had demonstrated a statistically significant incremental improvement in overall survival in patients with metastatic malignancies, and had been approved for marketing in the United States. Our observations are relevant internationally (and not just in the United States) as many jurisdictions may use the same clinical trials to support drug approval.

To estimate the number of life‐years that could potentially be saved for every year by which time from drug discovery to approval and funding could be reduced for these selected drugs (if all relevant patients were treated once the drug was approved), we multiplied the incremental improvement in median survival by the number of patients in North America 48, 49 and worldwide 50, 51 dying annually from the relevant malignancy, and then multiplied this by the estimated time from drug discovery until approval.

Where tumor subgroups were involved in the relevant study, death rates for the tumor and potential therapy benefits were adjusted based on published estimates of subgroup size.

NSCLC was calculated as 85% of all lung cancers, with squamous cell lung cancer comprising 25% and nonsquamous NSCLC 60% of all lung cancers 52. HER2‐positive breast cancer was calculated as 20% of all breast cancers 53. Colorectal cancers expressing EGFR by immunohistochemistry were calculated as 97% of all colorectal cancers 54, 55. HER2‐positive gastric cancer was calculated as 21% of all gastric cancers 32. Among malignant melanomas, about 50% are *BRAF*‐mutant 43. More than 90% of head and neck cancers are squamous cell 56.

We used date of initial US filing of a patent application as a surrogate for date of drug discovery. For drugs missing patent information, we instead used date of first synthesis, date of first publication on the agent, or (for some monoclonal antibodies) date that use of a similar antibody against the target was reported as being effective in animal models. We used date of approval by the US FDA as a surrogate for date of worldwide approval. This would generally underestimate the potential impact of gains in life‐years worldwide as US approval on average occurs earlier than approval in many other countries 57.

In Table 1 **,** we present the estimated life‐years potentially gained in North America (USA plus Canada) and worldwide per year of acceleration of drug approval and payer funding for the 21 therapies in 11 malignancies from our illustrative examples. The gain in median overall survival across studies ranged from 0.12 to 1.31 years (median, 0.31 years). Across these examples, the median potential gain in life‐years per year of acceleration of drug approval and payer funding would have been 5932 per example in North America and 79,920 worldwide if all relevant patients dying of the malignancy had been treated with the new agent and if all clinical trial results accurately reflected “real‐world” outcomes.

**Table 1:** Life‐years potentially gained per year of acceleration of drug approval for selected drugs

			No. patients per year potentially eligible for the therapy^a^		Life‐years potentially gained per year of acceleration of drug approval If all relevant patients were treated^b^	
Malignancy	Therapy (reference)	Median survival gain (years)	North America	Worldwide	North America	Worldwide
NSCLC	Erlotinib 21	0.17	152,794	1,351,418	25,975	229,741
NSCLC (nonsquamous)	Bevacizumab 22	0.17	95,559	953,941	16,245	162,170
NSCLC (squamous)	Nivolumab 23	0.27	44,941	397,474	12,134	107,318
Breast	Eribulin 24	0.21	45,529	521,900	9561	109,599
Breast (HER2+ve)	Trastuzumab 25	0.40	9106	104,371	3643	41,742
Breast (HER2+ve)	Trastuzumab emtansine 26	0.48	9106	104,371	4371	50,103
Breast (HER2+ve)	Pertuzumab 27	1.31	9106	104,371	11,929	136,737
Colorectal	Bevacizumab 28	0.39	59,100	693,900	23,248	270,621
Colorectal	Oxaliplatin 29	0.38	59,100	693,900	22,652	263,682
Colorectal	Regorafinib 30	0.12	59,100	693,900	7153	83,268
Colorectal (EGFR+ve)	Cetuximab 31	0.13	57,823	673,086	7517	87,501
Gastric (HER2+ve)	Trastuzumab 32	0.23	2748	151,852	632	34,926
Head/Neck (squamous)	Cetuximab 33	0.23	8926	199,800	2053	45,954
Prostate	Cabazitaxel 34	0.20	33,480	307,500	6,696	61,500
Prostate	Enzalutamide 35	0.40	33,480	307,500	13,392	123,000
Prostate	Abiraterone 36	0.38	33,480	307,500	12,722	116,850
Prostate	Sipuleucel‐T 37	0.36	33,480	307,500	12,053	110,700
Renal	Temsirolimus 38	0.30	15,610	116,000	4683	34,800
Renal	Sunitinib 39	0.38	15,610	116,000	5932	44,080
Renal^c^	Sorafenib 40	0.29	15,610	116,000	4527	33,640
Melanoma	Ipilumumab 41	0.31	10,760	46,000	3336	14,260
Melanoma (BRAF‐mutant)	Vemurafenib 42	0.33	5380	23,000	1775	7590
Melanoma (BRAF‐wild type)	Nivolumab 43	0.42	5380	23,000	2260	9660
Myeloma	Pomalidomide 44	0.39	12,400	72,000	4836	28,080
Myeloma	Bortezomib 45	1.11	12,490	72,000	13,864	79,920
Hepatocellular	Sorafenib 46	0.23	24,050	745,500	5532	171,465
Cervix	Bevacizumab 47	0.31	4400	265,700	1364	82,367
Median		0.31	15,610	265,700	5932	79,920
Cumulative (all sites/drugs combined)					240,085	2,541,274

a No. patients dying per year.

b Median survival gain (years) x no. patients dying per year.

c Control patients censored at time of cross‐over.

## Adjustment for Factors that Would Reduce “Real‐World” Benefit

“Real‐world” evidence suggests survival gains equivalent to those seen in clinical trials for some agents 58, but not others 59. Furthermore, many patients might not be treated even if a drug were approved and payer‐funded. Patients might elect not to proceed with treatment, even if it were offered, and the probability of a physician proposing treatment with the agent might be impacted by the physician's perception of the efficacy, cost‐effectiveness, and toxicity of the drug, by the availability of an effective alternative, and by the patient's performance status, comorbidities, etc. For example, in the province of Ontario, Canada, only 24% of patients with metastatic NSCLC receive approved, government‐funded systemic therapies 60, and there is much less access to effective new therapies in developing countries than in wealthier countries 18. Overall, access to new therapies varies widely across drugs and across countries. Oncology drugs currently account for the highest dollar share of worldwide sales of any class of agents, with one of the most rapid rates of sales growth 61, but we were unable to find any firm data on proportion of patients receiving therapy across the spectrum of new agents. However, even if the proportion treated is low, there could still be substantial numbers of life‐years saved by faster access to these effective new therapies.

## Life‐years Lost from Drug Discovery Until Approval

Table 2 shows life‐years potentially lost from drug discovery or patent application until approval, had all relevant patients been treated. The median time from discovery/patent application until approval was 12 (range, 6.1–23.3) years for our illustrative examples. The median number of life‐years lost worldwide between time of drug discovery and approval was 1,020,900 per example (range, 51,612–6,143,791). As noted above, the actual number would be substantially lower than this as many relevant patients would not have been treated even if the drug were available, but even if the proportion who would have been treated was very low, the number of life‐years lost from drug discovery to approval would nevertheless be expected to be high for many of these agents.

**Table 2:** Number of life‐years potentially lost in North America and worldwide between time of drug patent application and drug approval

					Life‐years lost from patent to approval	
Malignancy	Therapy	US Patent application date or drug discovery (reference)	FDA approval date (reference)	Patent to approval, years	North America	Worldwide
NSCLC	Erlotinib	05‐1996 94	11‐2004 95	8.5	220,790	1,952,795
NSCLC (nonsquamous)	Bevacizumab	05‐1995 96	10‐2006 97	11.4	185,193	1,848,738
NSCLC (squamous)	Nivolumab	2002^a^ ^98^	03‐2015 99	13	157,739	1,395,137
Breast	Eribulin	04‐2001 100	11‐2010 101	9.6	91,788	1,052,150
Breast (HER2+ve)	Trastuzumab	1990^b^ ^102^	09‐1998 103	8	29,138	334,015
Breast (HER2+ve)	Trastuzumab emtansine	10‐2004 104	02‐2013 105	8.3	36,276	415,849
Breast (HER2+ve)	Pertuzumab	06‐2000 106	06‐2012 107	12	143,157	1,640,853
Colorectal	Bevacizumab	05‐1995 96	02‐2004 97	8.8	204,582	2,381,465
Colorectal	Oxaliplatin	10‐1980^c^ ^108^	01‐2004 109	23.3	527,787	6,143,791
Colorectal	Regorafinib	01‐2000 110	02‐2012 111	12.1	86,554	1,007,543
Colorectal (EGFR‐+ve)	Cetuximab	09‐1988 112	02‐2004 113	15.4	115,760	1,347,512
Gastric (HER2+ve)	Trastuzumab	1990^b^ ^102^	10‐2010 114	20	12,645	698,515
Head/Neck (squamous)	Cetuximab	09‐1988 112	11‐2011 113	23.2	47,630	1,066,133
Prostate	Cabazitaxel	11‐1993 115	06‐2010 116	16.6	111,154	1,020,900
Prostate	Enzalutamide	05‐2006 62	08‐2012 63	6.3	84,370	774,900
Prostate	Abiraterone	09‐1994 117	04‐2011 118	16.6	211,192	1,939,710
Prostate	Sipuleucel‐T	09‐1998 119	04‐2010 120	11.6	139,815	1,284,120
Renal	Temsirolimus	04‐1994 121	05‐2007 122	13.1	61,347	455,880
Renal	Sunitinib	12‐1999^d^ ^64^	01‐2006 65	6.1	36,184	268,888
Renal^e^	Sorafenib	02‐1999 66	12‐2005 67	6.8	30,783	228,752
Melanoma	Ipilumumab	1997^f^ ^98^	03‐2011 123	14	46,698	199,640
Melanoma (RAF‐mutant)	Vemurafenib	12‐2004 68	08‐2011 69	6.8	12,073	51,612
Melanoma (RAF‐wild type)	Nivolumab	2002^a^ ^98^	12‐2014 99	12	27,115	115,920
Myeloma	Pomalidomide	07‐1996 124	02‐2013 125	16.6	80,278	466,128
Myeloma	Bortezomib	05‐1995 126	06‐2008 127	13.1	181,617	1,046,952
Hepatocellular	Sorafenib	02‐1999 66	11‐2007 67	8.8	48,677	1,508,892
Cervix	Bevacizumab	05‐1995 96	08‐2014	19.3	26,325	1,589,683
Median				12	80,278	1,020,900
Cumulative (all sites)					2,956,667	31,537,958

a Demonstration that PD‐L1 expression in mouse tumors confers immune resistance and that human tumors have high expression of PD‐L1.

b Creation of trastuzumab.

c First published report on oxaliplatin.

d Patent priority date.

e Control patients censored at time of cross‐over.

f Treatment of mouse tumors with anti‐CTLA‐4.

For the 27 drug‐tumor pairs in our illustrative examples, the FDA approved the drug 0.17–6.5 (median, 1.1) years prior to publication of the phase III trial results for 18 drugs, and 0–3.17 (median, 0.33) years after publication of the phase III data for nine drugs. Hence, the major factor determining time to drug approval was the time required for preclinical and clinical assessments rather than the review time required by regulatory agencies.

## Life‐Years Saved if Average Time from Drug Discovery to Approval and Payer Coverage were Reduced to 5 years

**Figure 1:**
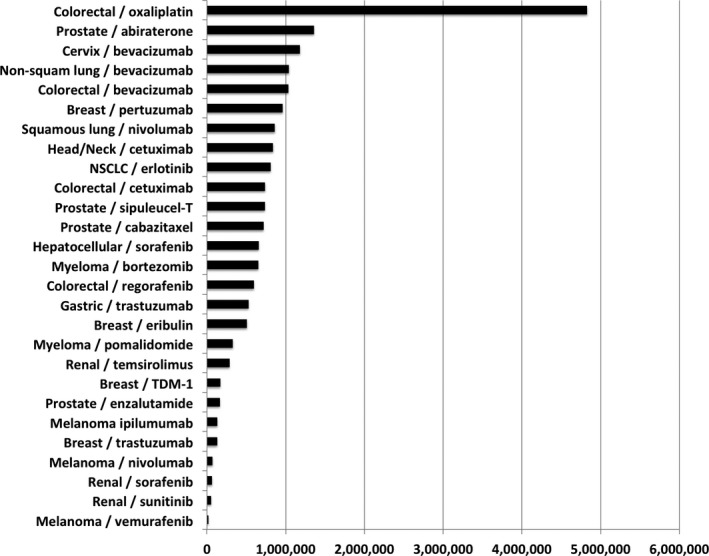
Cumulatively, across illustrative examples, more than 19,000,000 life‐years could potentially have been saved worldwide if time from drug discovery to approval for these agents had been reduced to 5 years, or more than 1,900,000 if (for example) only 10% of all relevant patients were to be treated.

As noted previously, while the length of time between drug discovery and approval lengthened progressively from the 1960s to the late 1990s, it has more recently begun to shorten for some agents 1, 2, 3, 18. Recent experience indicates that it could be feasible to reduce the time between drug discovery and approval to around 4–7 years 8, 9, 10, 11, 18, 62, 63, 64, 65, 66, 67, 68, 69. The median number of life‐years potentially saved by reducing the time between drug discovery and approval/funding to 5 years per example would have been 43,981 for North America (range, 3195–414,532) and would have been 523,890 worldwide (range, 13,662–4,825,381) (Fig. 1), if all relevant patients were to be treated.

## Discussion

Our analysis has the limitations that are inherent in any modeling study. However, we are also aware of five qualitative criticisms regarding the themes discussed in this manuscript. The first is that the data presented are of little ultimate importance as there are no feasible solutions that could speed drug development. The second is that many patients would not be able to access the new drug(s) even if they were approved, so correcting delays in approval would not accomplish much. The third is that important drug toxicity may be missed if clinical research approaches are changed. The fourth is that our work constitutes an unjustified attack on the FDA and other regulators. The fifth is that many investigational agents prove to be ineffective, and we do not take that into account.

However, these critiques, while each having some validity, do not diminish the importance of the themes presented. First, the nihilistic view that there are no feasible solutions that could speed drug development is not correct. Importantly, we will not solve the problem if we do not try. There will be a greater collective willingness to try to solve the problem if there is broad societal awareness that a problem actually does exist. The data we present here help illustrate how important this problem is. In addition, we and others have identified several pragmatic, feasible steps that could help 12, 13, 14, 15, 16, and we reiterate that the US FDA has taken a major step in the right direction with “breakthrough drug” approaches 4 that have markedly accelerated approval for selected effective agents. Breakthrough drug approval is a very important first step, but there are several other feasible value‐added steps that could pay important dividends.

We have no major issue with the approaches taken in assessment of therapies for nonlethal conditions, but for lethal diseases, we need “progress‐centered regulation,” with the primary objective being the rapid, affordable identification of new, effective agents. The launching of the “Moonshot” Cancer Campaign 70 was an important step in this direction.

One example of a step that can be improved is preclinical toxicology. Expensive preclinical toxicology (that takes months or years to complete prior to initiation of any clinical trials) can often be of limited value: It either predicts the obvious (e.g., that the drug will cause myelosuppression) or predicts toxicity that does not occur in humans or it misses things that end up being important. There are relatively few examples of preclinical toxicology assessments providing an early warning about important toxicity that might not have been anticipated from the drug mechanism of action or from toxicity of similar classes of agents already in clinical use. The solution for this part of the problem has already been tested and demonstrated to work: It has been shown that if one rapidly and inexpensively defines the dose of the agent that kills 10% of rodents (the LD10), then it is generally safe to use 1/10 the LD10 as the initial starting dose in clinical phase I trials 71, 72.

Once preclinical data are available, it can take months or years and hundreds of separate steps and processes (many with little added value) to take a clinical trial from initial concept to full activation. This needs to be markedly streamlined and accelerated 73, 74, and the US National Cancer Institute has implemented processes to begin to address this issue 75.

It can take months (or longer) and substantial resources for IRB approval and activation at each participating site, but, in the age of therapies targeting uncommon mutations, each site may accrue few or no patients after they have gone to the time and expense of activating the trial. What we need is “just‐in‐time” trial activation: If an accredited investigator identifies an eligible patient, they should be able to go online and immediately activate a trial that has already been approved elsewhere by an accredited IRB. That way, there is no delay in trial access and no wasted effort and expense activating a trial for which no patients are subsequently found at the individual institution 12, 13, 14, 15, 16. Assessments of this approach have been initiated 76.

Excessively restrictive exclusion criteria need to be addressed so that clinical trials are not unnecessarily slowed by inability to rapidly identify eligible patients, and there has been recent progress on this front as well 77.

Patient selection for a trial should be possible using a research laboratory biomarker rather than requiring the many months and large expense of CLIA certification or Investigational Device approval for the biomarker before the trial can be launched and before one has any clinical data to indicate whether or not the biomarker will actually be a useful predictor of drug efficacy 12, 13, 14, 15, 16. Use of research laboratory biomarkers to guide clinical trial accrual has proven to be feasible and effective 78.

We need to right size the currently massively excessive documentation that drives a large part of the rapidly escalating cost of clinical research, consumes hundreds of hours of investigator and research staff time and that often adds little value 12, 13, 14, 15, 16. The cost and complexity of all the “paperwork,” which is often not adequately reimbursed, is one important reason more oncologists do not participate in clinical trials. Work is underway to facilitate direct dumping of data from electronic medical records into research databases.

We need to rationalize the reporting of toxicity to reduce the huge effort that goes into reporting minor toxicity and to reduce the time and expense of producing and reviewing toxicity reports that often report once more a toxicity that is already well known or that present as possible toxicity events that are much more likely to be related to the underlying malignancy. To help address this issue, the FDA is currently working with pharmaceutical companies to rationalize the reporting of toxicity data. Furthermore, pragmatic postmarketing surveillance mechanisms have been proposed to monitor real‐world drug toxicity (and efficacy) and to replace in part the extensive, expensive detailed documentation of minor toxicities currently required during early clinical trials 16.

We need to reduce the rapidly escalating burden of overly restrictive privacy regulation, and we need to rationalize consent and reconsent processes 12, 13, 14, 15, 16.

Finally, in the age of massive computer databases and robust correlative studies, we need to be able to use real‐world data to gain approvals or add indications. A nascent example of such an approval is that of the checkpoint inhibitor pembrolizumab that was recently approved by the FDA for all solid tumors with high microsatellite instability (MSI‐H) 79. This approval went forward because of high response rates in these tumors. Importantly, it was in part based on retrospective data mining and correlative studies. The alterative would have been for the FDA to request a prospective study across tumor types, and this would have delayed approval and hence drug availability for years.

In summary, we have the tools to fix the problem. We just need to have the collective will to do so. Quantifying the size of the problem will hopefully help motivate change.

With respect to the question regarding drug pricing limiting drug availability and hence the usefulness of speeding discoveries, it is an undeniable disturbing fact that many patients both in North America and worldwide are unable to access effective new drugs even if they are approved. A life‐year saved is every bit as important if it is gained by faster access to drug payment as if it is gained by faster drug approval. However, faster drug approval is an essential component of better drug access. Outside of a clinical trial (and fewer than 5% of North American adult cancer patients are able to enroll on a clinical trial 80), regulatory approval of the drug is generally a prerequisite to broad access. Other factors such as drug funding and access to medical care may be of little consequence if the drug is not approved. Furthermore, the longer it takes the effective drug to get approved, the higher the likely price, as investors attempt to recoup expenses generated over multiple years and as duration of patent protection shrinks.

Once the drug is approved, rapidly escalating drug prices contribute to the inability of patients and healthcare systems to afford drug access. Drug development costs have been rising much faster than inflation, with little evidence that these increased costs translate into enhanced patient safety or better clinical trials 15, 81. These rapidly rising drug development costs are driven in part by the same accelerating regulatory complexity that delays drug approval. For example, from 1999 to 2005, unique investigational study procedures grew by 6.5% each year, procedural frequency rose by 8.7% annually, and the number of eligibility criteria per protocol tripled 82. Additionally, in phase I trials conducted between 2004 and 2007, an average of 45 safety monitoring processes/events were mandated in the first four weeks of the trial 83, while between 2009 and 2012, this had risen to a mean of 105 processes/events 84. It has historically taken a median of 370–500 distinct processes and 26 months to take a cooperative group trial from initial concept to activation 73, 74. Several separate phase I‐III clinical trials may be required for drug approval 85, and these studies are often performed sequentially, with similar delays encountered with each sequential step.

The steps we have proposed above to speed drug development would also have the potential to markedly reduce drug development costs. If these costs could be reduced, savings could be invested instead in testing of other new ideas, and this could directly speed progress.

While drug development costs are not the only factor driving an explosion in drug prices, they are a major contributing factor. If drug development costs can be brought down, then there would be at least the potential to reduce drug prices without discouraging investment in new drug development. If prices can be brought down, this would translate into improved cost‐effectiveness, better value for money, perhaps more willingness of physicians to prescribe these agents, and more rapid approval of payer coverage of effective new therapies. More rapid payer coverage would mean more life‐years saved. Reducing drug prices by reducing drug development costs is particularly crucial if the benefits of new effective therapies are to be made available to patients in less wealthy countries where access to new therapies is currently often poor 18.

The third critique of the proposals herein is that important drug toxicity may be missed if clinical research approaches are changed. For this reason, we agree that effective regulation is essential. Current clinical research regulatory and methodological approaches are intended to ensure safety and informed consent for study participants, to clarify risks and toxicities, to guarantee an appropriate level of safety for patients treated once a new agent is approved, and to ensure that agents do not get approved unless they add value. However, while it is currently felt that a price of $50,000–$250,000 per life‐year saved would be reasonable for a new drug 86, for participants in clinical trials, the cost per life‐year saved by increasingly complex clinical research regulation is in the millions of dollars 15. Furthermore, there has been little evidence that toxicity that was missed pre‐approval has been a major problem with the overwhelming majority of anticancer drugs, including those agents undergoing accelerated approval 87, and one may question if it is ethical to pursue randomized trials to more fully assess toxicity when it has already been established that one arm of a trial is clearly more effective than the other 88. Unquestionably, regulation is important and toxicity is important. Postmarketing surveillance of new agents is also important, and there is the potential for pragmatic ways to do this more effectively 16. There has also been successful implementation of clinical trials using greatly simplified, low cost designs that could facilitate ongoing postmarketing assessment of recently approved new therapies 89, 90. Overall, is it really justifiable to delay access to all effective new agents for lethal diseases based on the possibility that an important toxicity might initially be missed for a small proportion of patients being treated with a small proportion of these drugs?

The fourth common concern has been that our work constitutes an unjustified attack on the FDA and other regulators. Let us stress that this is far more than just a problem with the regulators. Clinical researchers, institutions, sponsors, institutional review boards, clinical research organizations, a number of government agencies outside of the FDA, and others all contribute to the problem in important ways, and all need to do their part by improving their efficiency, performance, and commitment to progress. From our discussions with people from both the FDA and Health Canada, we know that they know there are problems, and we know that they want as much as anyone to see these problems fixed. We all need to work together to fix them. As noted above, recent “breakthrough drug” approaches 4, permitting rapid approval of effective agents without phase III trials, are a major step in the right direction, and the enactment by the US Congress of the 21st Century Cures Act in December 2016 has the potential to help substantially 91. However, some of these important steps are facing opposition from some individuals and groups outside of regulatory bodies 92, and it is highly important that we support regulators worldwide in the drive to facilitate development of effective new anticancer drugs.

Finally, a fifth critique of the themes presented herein is that the importance of rapid, cost‐effective drug assessment is diminished by the fact that only a minority of new experimental agents prove effective 93. However, we believe that it is as important to rapidly identify ineffective agents as it is to quickly recognize effective agents. Inefficient studies of agents that ultimately prove to be ineffective compete for patients and other resources with studies of agents that ultimately prove to be effective: This slows progress. The suggestions herein should not serve to lower the bar so that ineffective drugs may be approved. Their purpose is to improve the efficiency and cost‐effectiveness of the entire drug development process so that we can rapidly and inexpensively identify those drugs that work and those drugs that do not work.

In summary, improving the efficiency of new drug development and decreasing the regulatory burden could translate into large benefits for patients, while the potential reduction in drug development costs is in everyone's best interest.

## Conflict of Interest

Dr. D. J. Stewart has received advisory board fees from Amgen, Roche Canada, Pfizer Canada, Boehringer Ingleheim Canada, and Novartis Canada, honoraria from Pfizer Canada and from the International Association for the Study of Lung Cancer, research funding from Celgene, Pfizer Canada and Roche Canada, and is Lead Advisor on the Life‐Saving Therapies Network. J.‐P. Bradford is President of Bradford Bachinski Limited, CEO of the Life‐Saving Therapies Network, cochair of the Advocacy Committee of Lung Cancer Canada, a member of the Cancer Care Advisory Committee of the Ottawa Regional Cancer Foundation, and a member of the Research Advisory Committee of the Canadian Partnership Against Cancer. Dr. R. Kurzrock has research funds from Incyte, Genentech, Merck Serono, Foundation Medicine, Guardant, Sequenom and Pfizer, as well as consultant fees from Acutate Therapeutics, XBiotech and Loxo, speaker fees from Roche, and an ownership interest in CureMatch Inc. Dr. J. H. Schiller is President of Free to Breathe, has done consulting and participated in advisory boards for Synta, Vertex, Genentech, Clovis, Biodesix, AVEO, Eisai, AbbVie, Lilly, Merck, and AstraZeneca, and has participated in clinical research with Clovis, AbbVie, Astex, Jannsen, Genentech, Synta, Sorono/EMD, Pfizer, PUMA, and Johnson & Johnson. Dr. G. Batist has received support for research from Pfizer and works in an academic‐industry Consortium that includes Merck, Roche, Pfizer, Amgen, Bayer, Novartis. Dr. Paul Wheatley‐Price is President of Lung Cancer Canada.

## References

[1] Dickson, M. , and J. P. Gagnon . 2004 The cost of new drug discovery and development. Discov. Med. 4:172–179. 20704981

[2] Pammolli, F. , L. Magazzini , and M. Riccaboni . 2011 The productivity crisis in pharmaceutical R&D. Nat. Rev. Drug. Discov. 10:428–438. 21629293 10.1038/nrd3405

[3] Langreth, R. , and O. Staley . 2015 Faster FDA spurs cancer breakthroughs. *Bloomberg Business* Available at http://www.bloomberg.com/news/articles/2015-03-24/faster-fda-spurs-cancer-breakthroughs-as-drug-approvals-surge. March 24.

[4] Horning, S. J. , D. A. Haber , W. K. Selig , S. P. Ivy , S. A. Roberts , J. D. Allen , et al. 2013 Developing standards for breakthrough therapy designation in oncology. Clin. Cancer Res. 19:4297–4304. 23719260 10.1158/1078-0432.CCR-13-0523 PMC3745545

[5] Flashback: FDA Drug Approvals 2013. Drugscom. Available at http://www.drugs.com/slideshow/new-and-unique-drugs-approved-in-2013-1071#slide-1 (accessed July 05, 2015).

[6] Novel new drugs 2014 *. US Food and Drug Administration Center for Drug Evaluation and Research* Available at http://www.fda.gov/downloads/Drugs/DevelopmentApprovalProcess/DrugInnovation/UCM430299.pdf (accessed July 05, 2015).

[7] Great Expectations ‐ 10 hot drug approvals for 2015. *Drugscom* Available at http://www.drugs.com/slideshow/great-expectations-the-10-most-hotly-anticipated-drugs-of-2015-1142 (accessed July 05, 2015).

[8] Enantiomerically pure aminoheteroaryl compounds as protein kinase inhibitors US 7858643 B2. Avilable at http://wwwgoogleje/patents/US7858643 (accessed July 05, 2015).

[9] Compounds and compositions as protein kinase inhibitors US 8039479 B2. Available at http://wwwgooglecom/patents/US8039479 (accessed July 05, 2015).

[10] FDA approval for crizotinib. Available at http://wwwcancergov/about-cancer/treatment/drugs/fda-crizotinib (accessed July 05, 2015).

[11] FDA approval of ceritinib. Available at http://wwwcancergov/about-cancer/treatment/drugs/fda-ceritinib (accessed July 05, 2015).

[12] Stewart, D. J. , G. Batist , H. M. Kantarjian , J. P. Bradford , J. Schiller , and R. Kurzrock . 2015 The urgent need for clinical research reform to permit faster, less expensive access to new therapies for lethal diseases. Clin. Cancer Res. 21:4561–4568. 26473192 10.1158/1078-0432.CCR-14-3246

[13] Stewart, D. J. , and J. P. Bradford . 2015 To benefit from new cancer drugs, reform the regulatory regime. *Globe and Mail* Available at http://www.theglobeandmail.com/globe-debate/to-benefit-from-new-cancer-drugs-reform-the-regulatory-regime/article22696420/

[14] Stewart, D. J. , and R. Kurzrock . 2009 Cancer: the road to Amiens. J. Clin. Oncol. 27:328–333. 19064964 10.1200/JCO.2008.18.9621

[15] Stewart, D. J. , S. N. Whitney , and R. Kurzrock . 2010 Equipoise lost: ethics, costs, and the regulation of cancer clinical research. J. Clin. Oncol. 28:2925–2935. 20406924 10.1200/JCO.2009.27.5404

[16] Stewart, D. J. , and G. Batist . 2014 Redefining cancer: a new paradigm for better and faster treatment innovation. J. Popul. Ther. Clin. Pharmacol. 21:e56–e65. 24671868

[17] Stewart, D. J. , and R. Kurzrock . 2013 Fool's gold, lost treasures, and the randomized clinical trial. BMC Cancer 13:193. 23587187 10.1186/1471-2407-13-193 PMC3639810

[18] Aitken, M. 2017 Global oncology trends 2017: advances, complexity and cost. QuntilesIMS Institute, Parsippany, NJ.

[19] Rovere, M. , and B. J. Skinner . 2012 Acess delayed, accessed denied 2012: waiting for new medicines in Canada. Available at http://wwwfraserinstituteorg

[20] Access to new medicines in public drug plans: Canada and comparable countries: 2015 annual report. Rx & D: Canada's Research‐Based Pharmaceutical Companies. 2015. Available at http://www.canadapharma.org/CMFiles/Our%20Industry/Industry%20Facts/RxD_6580_AccessToMedicinesReport_WEB.pdf

[21] Shepherd, F. A. , J. Rodrigues Pereira , T. Ciuleanu , E. H. Tan , V. Hirsh , S. Thongprasert , et al. 2005 Erlotinib in previously treated non‐small‐cell lung cancer. N. Engl. J. Med. 353:123–132. 16014882 10.1056/NEJMoa050753

[22] Sandler, A. , R. Gray , M. C. Perry , J. Brahmer , J. H. Schiller , A. Dowlati , et al. 2006 Paclitaxel‐carboplatin alone or with bevacizumab for non‐small‐cell lung cancer. N. Engl. J. Med. 355:2542–2550. 17167137 10.1056/NEJMoa061884

[23] Brahmer, J. , K. L. Reckamp , P. Baas , L. Crinò , W. E. Eberhardt , E. Poddubskaya , et al. 2015 Nivolumab versus docetaxel in advanced squamous‐cell non‐small‐cell lung cancer. N. Engl. J. Med. 373:123–135. 26028407 10.1056/NEJMoa1504627 PMC4681400

[24] Cortes, J. , J. O'Shaughnessy , D. Loesch , J. L. Blum , L. T. Vahdat , K. Petrakova , et al. 2011 Eribulin monotherapy versus treatment of physician's choice in patients with metastatic breast cancer (EMBRACE): a phase 3 open‐label randomised study. Lancet 377:914–923. 21376385 10.1016/S0140-6736(11)60070-6

[25] Slamon, D. J. , B. Leyland‐Jones , S. Shak , H. Fuchs , V. Paton , A. Bajamonde , et al. 2001 Use of chemotherapy plus a monoclonal antibody against HER2 for metastatic breast cancer that overexpresses HER2. N. Engl. J. Med. 344:783–792. 11248153 10.1056/NEJM200103153441101

[26] Verma, S. , D. Miles , L. Gianni , I. E. Krop , M. Welslau , J. Baselga , et al. 2012 Trastuzumab emtansine for HER2‐positive advanced breast cancer. N. Engl. J. Med. 367:1783–1791. 23020162 10.1056/NEJMoa1209124 PMC5125250

[27] Swain, S. M. , J. Baselga , S. B. Kim , J. Ro , V. Semiglazov , M. Campone , et al. 2015 Pertuzumab, trastuzumab, and docetaxel in HER2‐positive metastatic breast cancer. N. Engl. J. Med. 372:724–734. 25693012 10.1056/NEJMoa1413513 PMC5584549

[28] Hurwitz, H. , L. Fehrenbacher , W. Novotny , T. Cartwright , J. Hainsworth , W. Heim , et al. 2004 Bevacizumab plus irinotecan, fluorouracil, and leucovorin for metastatic colorectal cancer. N. Engl. J. Med. 350:2335–2342. 15175435 10.1056/NEJMoa032691

[29] Goldberg, R. M. , D. J. Sargent , R. F. Morton , C. S. Fuchs , R. K. Ramanathan , S. K. Williamson , et al. 2004 A randomized controlled trial of fluorouracil plus leucovorin, irinotecan, and oxaliplatin combinations in patients with previously untreated metastatic colorectal cancer. J. Clin. Oncol. 22:23–30. 14665611 10.1200/JCO.2004.09.046

[30] Grothey, A. , E. Van Cutsem , A. Sobrero , S. Siena , A. Falcone , M. Ychou , et al. 2013 Regorafenib monotherapy for previously treated metastatic colorectal cancer (CORRECT): an international, multicentre, randomised, placebo‐controlled, phase 3 trial. Lancet 381:303–312. 23177514 10.1016/S0140-6736(12)61900-X

[31] Jonker, D. J. , C. J. O'Callaghan , C. S. Karapetis , J. R. Zalcberg , D. Tu , H. J. Au , et al. 2007 Cetuximab for the treatment of colorectal cancer. N. Engl. J. Med. 357:2040–2048. 18003960 10.1056/NEJMoa071834

[32] Bang, Y. J. , E. Van Cutsem , A. Feyereislova , H. C. Chung , L. Shen , A. Sawaki , et al. 2010 Trastuzumab in combination with chemotherapy versus chemotherapy alone for treatment of HER2‐positive advanced gastric or gastro‐oesophageal junction cancer (ToGA): a phase 3, open‐label, randomised controlled trial. Lancet 376:687–697. 20728210 10.1016/S0140-6736(10)61121-X

[33] Vermorken, J. B. , R. Mesia , F. Rivera , E. Remenar , A. Kawecki , S. Rottey , et al. 2008 Platinum‐based chemotherapy plus cetuximab in head and neck cancer. N. Engl. J. Med. 359:1116–1127. 18784101 10.1056/NEJMoa0802656

[34] de Bono, J. S. , S. Oudard , M. Ozguroglu , S. Hansen , J. P. Machiels , I. Kocak , et al. 2010 Prednisone plus cabazitaxel or mitoxantrone for metastatic castration‐resistant prostate cancer progressing after docetaxel treatment: a randomised open‐label trial. Lancet 376:1147–1154. 20888992 10.1016/S0140-6736(10)61389-X

[35] Scher, H. I. , K. Fizazi , F. Saad , M. E. Taplin , C. N. Sternberg , K. Miller , et al. 2012 Increased survival with enzalutamide in prostate cancer after chemotherapy. N. Engl. J. Med. 367:1187–1197. 22894553 10.1056/NEJMoa1207506

[36] Fizazi, K. , H. I. Scher , A. Molina , C. J. Logothetis , K. N. Chi , R. J. Jones , et al. 2012 Abiraterone acetate for treatment of metastatic castration‐resistant prostate cancer: final overall survival analysis of the COU‐AA‐301 randomised, double‐blind, placebo‐controlled phase 3 study. Lancet Oncol. 13:983–992. 22995653 10.1016/S1470-2045(12)70379-0

[37] Higano, C. S. , P. F. Schellhammer , E. J. Small , P. A. Burch , J. Nemunaitis , L. Yuh , et al. 2009 Integrated data from 2 randomized, double‐blind, placebo‐controlled, phase 3 trials of active cellular immunotherapy with sipuleucel‐T in advanced prostate cancer. Cancer 115:3670–3679. 19536890 10.1002/cncr.24429

[38] Kwitkowski, V. E. , T. M. Prowell , A. Ibrahim , A. T. Farrell , R. Justice , S. S. Mitchell , et al. 2010 FDA approval summary: temsirolimus as treatment for advanced renal cell carcinoma. Oncologist 15:428–435. 20332142 10.1634/theoncologist.2009-0178 PMC3227966

[39] Motzer, R. J. , T. E. Hutson , P. Tomczak , M. D. Michaelson , R. M. Bukowski , S. Oudard , et al. 2009 Overall survival and updated results for sunitinib compared with interferon alfa in patients with metastatic renal cell carcinoma. J. Clin. Oncol. 27:3584–3590. 19487381 10.1200/JCO.2008.20.1293 PMC3646307

[40] Escudier, B. , T. Eisen , W. M. Stadler , C. Szczylik , S. Oudard , M. Staehler , et al. 2009 Sorafenib for treatment of renal cell carcinoma: Final efficacy and safety results of the phase III treatment approaches in renal cancer global evaluation trial. J. Clin. Oncol. 27:3312–3318. 19451442 10.1200/JCO.2008.19.5511

[41] Hodi, F. S. , S. J. O'Day , D. F. McDermott , R. W. Weber , J. A. Sosman , J. B. Haanen , et al. 2010 Improved survival with ipilimumab in patients with metastatic melanoma. N. Engl. J. Med. 363:711–723. 20525992 10.1056/NEJMoa1003466 PMC3549297

[42] McArthur, G. A. , P. B. Chapman , C. Robert , J. Larkin , J. B. Haanen , R. Dummer , et al. 2014 Safety and efficacy of vemurafenib in BRAF(V600E) and BRAF(V600K) mutation‐positive melanoma (BRIM‐3): extended follow‐up of a phase 3, randomised, open‐label study. Lancet Oncol. 15:323–332. 24508103 10.1016/S1470-2045(14)70012-9 PMC4382632

[43] Robert, C. , G. V. Long , B. Brady , C. Dutriaux , M. Maio , L. Mortier , et al. 2015 Nivolumab in previously untreated melanoma without BRAF mutation. N. Engl. J. Med. 372:320–330. 25399552 10.1056/NEJMoa1412082

[44] San Miguel, J. , K. Weisel , P. Moreau , M. Lacy , K. Song , M. Delforge , et al. 2013 Pomalidomide plus low‐dose dexamethasone versus high‐dose dexamethasone alone for patients with relapsed and refractory multiple myeloma (MM‐003): a randomised, open‐label, phase 3 trial. Lancet Oncol. 14:1055–1066. 24007748 10.1016/S1470-2045(13)70380-2

[45] San Miguel, J. F. , R. Schlag , N. K. Khuageva , M. A. Dimopoulos , O. Shpilberg , M. Kropff , et al. 2013 Persistent overall survival benefit and no increased risk of second malignancies with bortezomib‐melphalan‐prednisone versus melphalan‐prednisone in patients with previously untreated multiple myeloma. J. Clin. Oncol. 31:448–455. 23233713 10.1200/JCO.2012.41.6180

[46] Llovet, J. M. , S. Ricci , V. Mazzaferro , P. Hilgard , E. Gane , J. F. Blanc , et al. 2008 Sorafenib in advanced hepatocellular carcinoma. N. Engl. J. Med. 359:378–390. 18650514 10.1056/NEJMoa0708857

[47] Tewari, K. S. , M. W. Sill , H. J. 3rd Long , R. T. Penson , H. Huang , L. M. Ramondetta , et al. 2014 Improved survival with bevacizumab in advanced cervical cancer. N. Engl. J. Med. 370:734–743. 24552320 10.1056/NEJMoa1309748 PMC4010094

[48] Siegel, R. , J. Ma , Z. Zou , and A. Jemal . 2014 Cancer statistics, 2014. CA Cancer J. Clin. 64:9–29. 24399786 10.3322/caac.21208

[49] Statistics CCSsACoC . 2014 Canadian Cancer Statistics 2014. Toronto, ON.

[50] Torre, L. A. , F. Bray , R. L. Siegel , J. Ferlay , J. Lortet‐Tieulent , and A. Jemal . 2015 Global cancer statistics, 2012. CA Cancer J. Clin. 65:87–108. 25651787 10.3322/caac.21262

[51] Ferlay, J. , H. R. Shin , F. Bray , D. Forman , C. Mathers , and D. M. Parkin . 2010 Estimates of worldwide burden of cancer in 2008: GLOBOCAN 2008. Int. J. Cancer 127:2893–2917. 21351269 10.1002/ijc.25516

[52] What is non‐small cell lung cancer? Available at http://wwwcancerorg/cancer/lungcancer-non-smallcell/detailedguide/non-small-cell-lung-cancer-what-is-non-small-cell-lung-cancer (accessed June 22, 2015).

[53] Wolff, A. C. , M. E. Hammond , J. N. Schwartz , K. L. Hagerty , D. C. Allred , R. J. Cote , et al. 2007 American Society of Clinical Oncology/College of American Pathologists guideline recommendations for human epidermal growth factor receptor 2 testing in breast cancer. Arch. Pathol. Lab. Med. 131:18–43. 19548375 10.5858/2007-131-18-ASOCCO

[54] Spano, J. P. , C. Lagorce , D. Atlan , G. Milano , J. Domont , R. Benamouzig , et al. 2005 Impact of EGFR expression on colorectal cancer patient prognosis and survival. Ann. Oncol. 16:102–108. 15598946 10.1093/annonc/mdi006

[55] Yarom, N. , C. Marginean , T. Moyana , I. Gorn‐Hondermann , H. C. Birnboim , H. Marginean , et al. 2010 EGFR expression variance in paired colorectal cancer primary and metastatic tumors. Cancer Biol. Ther. 10:416–421. 20595818 10.4161/cbt.10.5.12610

[56] Gregoire, V. , J. L. Lefebvre , L. Licitra , and E. Felip . 2010 Squamous cell carcinoma of the head and neck: EHNS‐ESMO‐ESTRO Clinical Practice Guidelines for diagnosis, treatment and follow‐up. Ann. Oncol. 21(Suppl. 5):v184–v186. 20555077 10.1093/annonc/mdq185

[57] Downing, N. S. , J. A. Aminawung , N. D. Shah , J. B. Braunstein , H. M. Krumholz , and J. S. Ross . 2012 Regulatory review of novel therapeutics–comparison of three regulatory agencies. N. Engl. J. Med. 366:2284–2293. 22591257 10.1056/NEJMsa1200223 PMC3504361

[58] Harrison, L. D. , J. Zhang‐Salomons , M. Mates , C. M. Booth , W. D. King , and W. J. Mackillop . 2015 Comparing effectiveness with efficacy: outcomes of palliative chemotherapy for non‐small‐cell lung cancer in routine practice. Curr. Oncol. 22:184–191. 26089717 10.3747/co.22.2419 PMC4462528

[59] Booth, C. M. , and I. F. Tannock . 2014 Randomised controlled trials and population‐based observational research: partners in the evolution of medical evidence. Br. J. Cancer 110:551–555. 24495873 10.1038/bjc.2013.725 PMC3915111

[60] Sacher, A. G. , L. W. Le , A. Lau , C. C. Earle , and N. B. Leighl . 2015 Real‐world chemotherapy treatment patterns in metastatic non‐small cell lung cancer: Are patients undertreated? Cancer 121:2562–2569. 25891153 10.1002/cncr.29386

[61] Iervolino, A. , and L. Urquhart . 2017 Evaluate Pharma World Preview 2017, Outlook to 2022. Evaluate Ltd, London, UK.

[62] Diarylhydantoin compounds US 7709517 B2. Available at http://wwwgooglecom/patents/US7709517 (accessed June 21, 2015).

[63] FDA approval for enzalutamide. Available at http://wwwcancergov/about-cancer/treatment/drugs/fda-enzalutamide (accessed June 21, 2015).

[64] Methods of modulating c‐kit tyrosine protein kinase function with indolinone compounds US 20040002543 A1. Available at https://wwwgooglecom/patents/US20040002534?dq=patent+US+Ser+No+60/171,693&hl=en&sa=X&ei=VxqHVcbpOYyTyATU4YDoBg&ved=0CCwQ6AEwAg (accessed June 21, 2015).

[65] FDA approval for sunitinb malate. Available at http://wwwcancergov/about-cancer/treatment/drugs/fda-sunitinib-malate. (accessed June 21, 2015).

[66] Omega‐carboxyl aryl substituted diphenyl ureas as raf kinase inhibitors, United States Patent and Trademark Office, Patent Application No. 09257266. Available at http://wwwplainsiteorg/dockets/1pxc3i0ga/united-states-patent-and-trademark-office/omegacarboxy-aryl-substituted-diphenyl-ureas-as-raf-kinase-inhibitors/ (accessed June 21, 2015).

[67] FDA approval for sorafenib tosylate. Available at http://wwwcancergov/about-cancer/treatment/drugs/fda-sorafenib-tosylate (accessed online June 21, 2015).

[68] Compounds and methods for development of Ret modulators US 7504509 B2. Available at http://wwwgooglecom/patents/US7504509 (accessed June 21, 2015).

[69] FDA approval for vemurafenib. Available at http://wwwcancergov/about-cancer/treatment/drugs/fda-vemurafenib (accessed June 21, 2015).

[70] Kaiser, J. , and J. Couzin‐Frankel . 2016 Biomedical Research. Biden seeks clear course for his cancer moonshot. Science 351:325–326. 26797992 10.1126/science.351.6271.325

[71] Newell, D. R. , S. S. Burtles , B. W. Fox , D. I. Jodrell , and T. A. Connors . 1999 Evaluation of rodent‐only toxicology for early clinical trials with novel cancer therapeutics. Br. J. Cancer 81:760–768. 10555743 10.1038/sj.bjc.6690761 PMC2374299

[72] Newell, D. R. , J. Silvester , C. McDowell , and S. S. Burtles . 2004 The Cancer Research UK experience of pre‐clinical toxicology studies to support early clinical trials with novel cancer therapies. Eur. J. Cancer 40:899–906. 15120045 10.1016/j.ejca.2003.12.020

[73] Dilts, D. M. , A. B. Sandler , M. Baker , S. K. Cheng , S. L. George , K. S. Karas , et al. 2006 Processes to activate phase III clinical trials in a Cooperative Oncology Group: the Case of Cancer and Leukemia Group B. J. Clin. Oncol. 24:4553–4557. 17008694 10.1200/JCO.2006.06.7819

[74] Dilts, D. M. , A. Sandler , S. Cheng , J. Crites , L. Ferranti , A. Wu , et al. 2008 Development of clinical trials in a cooperative group setting: the eastern cooperative oncology group. Clin. Cancer Res. 14:3427–3433. 18519773 10.1158/1078-0432.CCR-07-5060 PMC2723742

[75] Abrams, J. S. , M. M. Mooney , J. A. Zwiebel , E. L. Korn , S. H. Friedman , S. R. Finnigan , et al. 2013 Implementation of timeline reforms speeds initiation of National Cancer Institute‐sponsored trials. J. Natl Cancer Inst. 105:954–959. 23776198 10.1093/jnci/djt137 PMC3699438

[76] Pharmatech . Just‐in‐time rapid enrollment. Available at http://pharmatechcom/services/just-in-time/ (accessed January 08, 2018).

[77] Kim, E. S. , S. S. Bruinooge , S. Roberts , G. Ison , N. U. Lin , L. Gore , et al. 2017 Broadening eligibility criteria to make clinical trials more representative: American society of clinical oncology and friends of cancer research joint research statement. J. Clin. Oncol. 35:3737–3744. 28968170 10.1200/JCO.2017.73.7916 PMC5692724

[78] Kim, E. S. , R. S. Herbst , I. I. Wistuba , J. J. Lee , G. R. Blumenschein , A. Tsao , et al. 2011 The BATTLE trial: personalizing therapy for lung cancer. Cancer Discov. 1:44–53. 22586319 10.1158/2159-8274.CD-10-0010 PMC4211116

[79] Lemery, S. , P. Keegan , and R. Pazdur . 2017 First FDA approval agnostic of cancer site ‐ when a biomarker defines the indication. N. Engl. J. Med. 377:1409–1412. 29020592 10.1056/NEJMp1709968

[80] Sateren, W. B. , E. L. Trimble , J. Abrams , O. Brawley , N. Breen , L. Ford , et al. 2002 How sociodemographics, presence of oncology specialists, and hospital cancer programs affect accrual to cancer treatment trials. J. Clin. Oncol. 20:2109–2117. 11956272 10.1200/JCO.2002.08.056

[81] DiMasi, J. A. , R. W. Hansen , and H. G. Grabowski . 2003 The price of innovation: new estimates of drug development costs. J. Health Econ. 22:151–185. 12606142 10.1016/S0167-6296(02)00126-1

[82] Getz, K. 2008 Protocol design trends and their effect on clinical trial performance. RAJ Pharma. (May):315–316.

[83] Craft, B. S. , R. Kurzrock , X. Lei , R. Herbst , S. Lippman , S. Fu , et al. 2009 The changing face of phase 1 cancer clinical trials: new challenges in study requirements. Cancer 115:1592–1597. 19165808 10.1002/cncr.24171 PMC2668727

[84] Kurzrock, R. , and D. J. Stewart . 2013 Compliance in early‐phase cancer clinical trials research. Oncologist 18:308–313. 23457001 10.1634/theoncologist.2012-0260 PMC3607528

[85] Milne, C. P. 2002 Orphan products–pain relief for clinical development headaches. Nat. Biotechnol. 20:780–784. 12148001 10.1038/nbt0802-780

[86] Ubel, P. A. , S. R. Berry , E. Nadler , C. M. Bell , M. A. Kozminski , J. A. Palmer , et al. 2012 In a survey, marked inconsistency in how oncologists judged value of high‐cost cancer drugs in relation to gains in survival. Health Aff. (Millwood) 31:709–717. 22492887 10.1377/hlthaff.2011.0251 PMC3821071

[87] Tsimberidou, A. M. , F. Braiteh , D. J. Stewart , and R. Kurzrock . 2009 Ultimate fate of oncology drugs approved by the us food and drug administration without a randomized Trial. J. Clin. Oncol. 27:6243–6250. 19826112 10.1200/JCO.2009.23.6018

[88] Kurzrock, R. , and D. J. Stewart . 2013 Equipoise abandoned? Randomization and clinical trials Ann. Oncol. 24:2471–2474. 24072520 10.1093/annonc/mdt358

[89] Ibrahim, M. F. K. , J. Hilton , S. Mazzarello , D. Fergusson , B. Hutton , A. Robinson , et al. 2017 A multi‐center pragmatic, randomized, feasibility trial comparing standard of care schedules of filgrastim administration for primary febrile neutropenia prophylaxis in early‐stage breast cancer. Breast Cancer Res. Treat. 168:371–379. 29214415 10.1007/s10549-017-4604-y

[90] Hilton, J. , S. Mazzarello , D. Fergusson , A. A. Joy , A. Robinson , A. Arnaout , et al. 2016 Novel methodology for comparing standard‐of‐care interventions in patients with cancer. J. Oncol. Pract. 12:e1016–e1024. 27650842 10.1200/JOP.2016.013474

[91] Burris, J. F. , and J. T. Puglisi . 2017 Impact of federal regulatory changes on clinical pharmacology and drug development: the common rule and the 21st century cures act. J. Clin. Pharmacol. https://doi.org/10.1002/jcph.1026. 10.1002/jcph.1026 28981164

[92] Winners and losers of the 21st Century Cures Act. Available at https://wwwstatnewscom/2016/12/05/21st-century-cures-act-winners-losers/ (accessed January 12, 2017).

[93] Hay, M. , D. W. Thomas , J. L. Craighead , C. Economides , and J. Rosenthal . 2014 Clinical development success rates for investigational drugs. Nat. Biotechnol. 32:40–51. 24406927 10.1038/nbt.2786

[94] Alkynyl and azido‐substituted 4‐anilinoquinazolines US 5747498 A. Available at http://wwwgooglecom/patents/US5747498 (accessed June 22, 2015).

[95] FDA approval for erlotinib hydrochloride. Available at http://wwwcancergov/about-cancer/treatment/drugs/fda-erlotinib-hydrochloride (accessed June 22, 2015).

[96] Avastin (Bevacizumab). Available at http://wwwcptechorg/ip/health/avastinhtml (accessed June 20, 2015).

[97] Avastin approval history. Available at http://wwwdrugscom/history/avastinhtml (accessed June 20, 2015).

[98] Pardoll, D. M. 2012 Immunology beats cancer: a blueprint for successful translation. Nat. Immunol. 13:1129–1132. 23160205 10.1038/ni.2392 PMC4659410

[99] Opdivo approval history. Available at http://wwwdrugscom/history/opdivohtml (accessed June 20, 2015).

[100] Eribulin . DrugBank. Available at http://wwwdrugbankca/drugs/db08871 (accessed June 20, 2015).

[101] Halavan approval status. Available at http://wwwdrugscom/history/halavenhtml (accessed June 20, 2015).

[102] Herceptin (trastuzumab) development timelines. Available at http://wwwgenecom/media/product-information/herceptin-development-timeline (accessed June 20, 2015).

[103] Trastuzumab product approval information ‐ licensing action 09/25/98. Available at http://wwwfdagov/Drugs/DevelopmentApprovalProcess/HowDrugsareDevelopedandApproved/ApprovalApplications/TherapeuticBiologicApplications/ucm080591htm (accessed June 21, 2015).

[104] Conjugates of maytansinoid Dm1 with antibody trastuzumab, linked through a non‐cleavable linker, and its use in the treatment of tumors. Available at https://wwwlensorg/lens/patent/EP_1689846_B1 (accessed June 21, 2015).

[105] Ado‐trastuzumab emtansine. Available at http://wwwfdagov/Drugs/InformationOnDrugs/ApprovedDrugs/ucm340913htm (accessed June 21, 2015).

[106] Humanized anti‐ErbB2 antibodies and treatment with anti‐ErbB2 antibodies US 6949245 B1. http://wwwgooglecomar/patents/US6949245 (accessed June 21, 2015).

[107] FDA approval for pertuzumab. Available at http://wwwcancergov/about-cancer/treatment/drugs/fda-pertuzumab#Anchor-MetastatiHER2 (accessed June 21, 2015).

[108] Kidani, Y. , M. Noji , and T. Tashiro . 1980 Antitumor activity of platinum(II) complexes of 1,2‐diamino‐cyclohexane isomers. Gan 71:637–643. 7227714

[109] FDA approval for oxaliplatin. Available at http://wwwcancergov/about-cancer/treatment/drugs/fda-oxaliplatin (accessed June 21, 2015).

[110] w‐Carboxyaryl substituted diphenyl ureas as raf kinase inhibitors US 7351834 B1. Available at http://wwwgooglecom/patents/US7351834 (accessed June 21, 2015).

[111] FDA approval for regorafenib. Available at http://wwwcancergov/about-cancer/treatment/drugs/fda-regorafenib (accessed June 21, 2015).

[112] Monoclonal antibodies specific to human epidermal growth factor receptor and therapeutic methods employing same US 6217866 B1. Available at http://wwwgooglecom/patents/US6217866 (accessed June 20, 2015).

[113] FDA approval for cetuximab. Available at http://wwwcancergov/about-cancer/treatment/drugs/fda-cetuximab#Anchor-Colorecta-60318 (accessed June 20, 2015).

[114] FDA approval for trastuzumab. Available at http://wwwcancergov/about-cancer/treatment/drugs/fda-trastuzumab#Anchor-Gastric (accessed June 21, 2015).

[115] Taxoid‐based compositions US 5438072 A. Available at http://wwwgoogleca/patents/US5438072 (accessed June 21, 2015).

[116] FDA approval for cabazitaxel. Available at http://wwwcancergov/about-cancer/treatment/drugs/fda-cabazitaxel (accessed June 21, 2015).

[117] 17‐substituted steroids useful in cancer treatment US 5604213 A. Available at http://wwwgooglecom/patents/US5604213 (accessed June 21, 2015).

[118] FDA approval for abiraterone acetate. Available at http://wwwcancergov/about-cancer/treatment/drugs/fda-abirateroneacetate (accessed June 21, 2015).

[119] Immunostimulatory compositions US 5976546 A. Available at https://wwwgooglecom/patents/US5976546 (accessed June 21, 2015).

[120] FDA approval for Sipuleucel‐T. Available at http://wwwcancergov/about-cancer/treatment/drugs/fda-sipuleucel-T (accessed June 21, 2015).

[121] Rapamycin hydroxyesters US 5362718 A. Available at http://wwwgooglecom/patents/US5362718 (accessed June 21, 2015).

[122] FDA approval for temsirolimus. Available at http://wwwcancergov/about-cancer/treatment/drugs/fda-temsirolimus (accessed June 21, 2015).

[123] Yervoy approval history. Available at http://wwwdrugscom/history/yervoyhtml (accessed June 21, 2015).

[124] Method of reducing TNF alpha levels with amino substituted 2‐(2,6‐dioxopiperidin‐3‐yl)‐1‐oxo‐ and 1,3‐dioxoisoindolines US 5635517 A. Available at http://wwwgooglecom/patents/US5635517 (accessed June 21, 2015).

[125] FDA approval for pomalidomide. Available at http://wwwcancergov/about-cancer/treatment/drugs/fda-pomalidomide (accessed June 21, 2015).

[126] Boronic ester and acid compounds, synthesis and uses US 6083903 A. Available at http://wwwgoogleca/patents/US6083903. (accessed June 21, 2015).

[127] FDA approval for bortezomib. Available at http://wwwcancergov/about-cancer/treatment/drugs/fda-bortezomib. (accessed June 22, 2015).

